# Paucisymptomatic post COVID-19 myocarditis in a young athlete during return to play workflow: possible usefulness of global longitudinal strain analysis

**DOI:** 10.1136/bcr-2023-255863

**Published:** 2024-01-12

**Authors:** Gabriele De Masi De Luca, Paola Papadia, Zefferino Palamà, Giovanni Coluccia

**Affiliations:** 1 Cardiology Unit, Hospital Cardinal G Panico, Tricase, Italy; 2 Department of Clinical Medicine, Public Health, Life and Environmental Sciences, University of L'Aquila, L'Aquila, Italy; 3 Cardiomed Medical Center, Maglie, Italy; 4 Villa Verde Private Hospital Srl, Taranto, Italy

**Keywords:** COVID-19, Cardiovascular medicine

## Abstract

A young competitive athlete undergoes the diagnostic investigations protocol before returning to competitive practice (return to play protocol) after COVID-19 infection. Despite the paucisymptomatic presentation of COVID-19 infection and the absence of relevant anomalies in standard first-level diagnostic investigations, echocardiographic examination findings especially speckle tracking analysis (global longitudinal strain) along with some clinical aspects suggested further second-level investigations eventually allowing the identification of inflammatory myocardial damage.

## Background

COVID-19 infection is phenotypically expressed mainly with respiratory symptoms, but it is known that it also has a tropism for different organs and systems such as cardiac muscular tissue.^1 2^ Several observational studies documented the presence of subclinical inflammatory status even in asymptomatic or paucisymptomatic patients, accurately detectable with the use of cardiac MRI, where standard echocardiographic analysis often does not demonstrate significant anomalies.^3^

In the recent pandemic period, myocarditis in the young athlete showed a certain benignity. However, although rare, myocarditis is a cause of death in young athletes to whom a more detailed diagnostic process is adopted before returning to competitive practice.^4^

The MRI is not often quickly available to evaluate a post-COVID athlete for a safe return to competitive activity, nor can it be considered a large-scale screening method.

Echocardiographic analysis in COVID-19-hospitalised patients showed that the left ventricular global longitudinal strain (GLS) is significantly reduced^5^ as well as in post-COVID paucisymptomatic patients regardless of clinical presentation.^6^

A competitive cyclist underwent the diagnostic investigations protocol before returning to competitive practice (return to play protocol) after COVID-19 infection.

Despite the paucisymptomatic presentation of COVID-19 infection and the absence of relevant anomalies in standard first-level diagnostic investigations, echocardiographic examination findings especially speckle tracking analysis along with some modest clinical aspects suggested further second-level investigations eventually allowing the identification of myocardial damage.

## Case presentation

A competitive cyclist in his 30s came to our attention after having contracted COVID-19 infection in May 2022. The course of the infection had been characterised by 48 hours of hyperpyrexia and 5 days of persistent coughing. COVID-19 test became negative on the 21st day.

**Figure 1:**
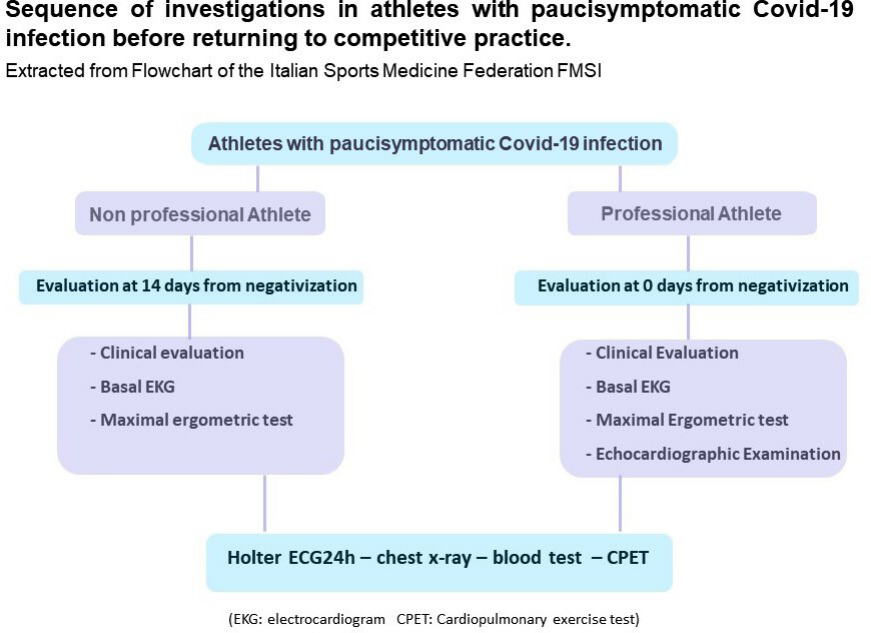
Sequence of investigations in athletes with paucisymptomatic COVID-19 infection before returning to competitive practice.

The patient accessed our diagnostic centre to undergo the investigations required by the ‘return to play’ protocol before returning to competitive sports (figure 1).

Despite having tried to remain active during the entire isolation period, the patient still reported fatigue and dyspnoea on moderate efforts.

The physical examination was unremarkable. Blood pressure was 120/75 mm Hg and heart rate was 91 beats per minute.

ECG and spirometry examination did not show significant abnormalities.

Negative inflammation (high-sensitivity C reactive protein) and myocardial damage markers (high-sensitivity troponin) were observed.

**Figure 2:**
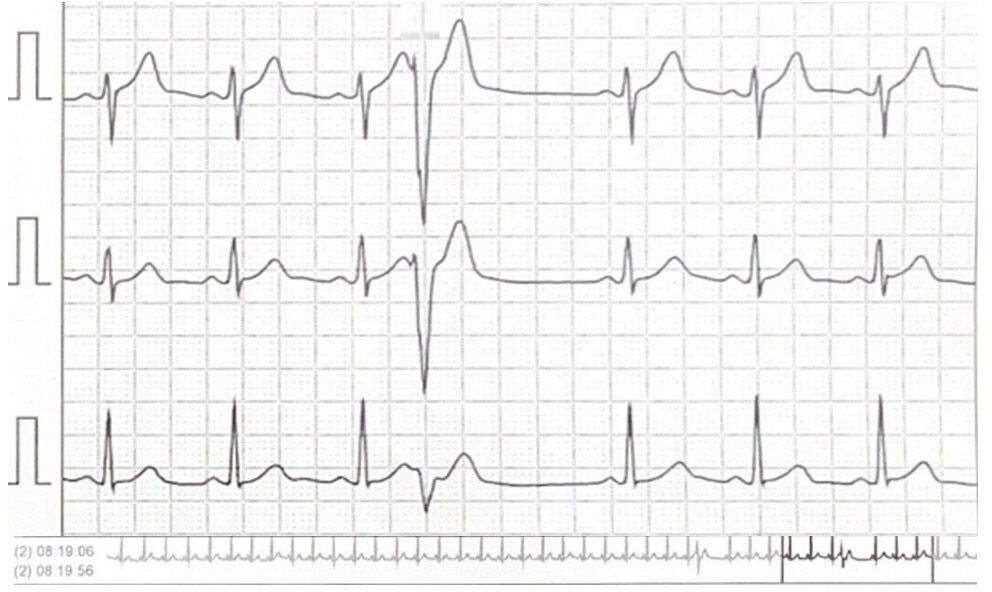
Holter-ECG tracing with an early premature ventricular complex.

The cycloergometer test was conducted up to stage 4 of Bruce protocol and showed no signs of myocardial distress, no induced relevant arrhythmias, rapid chronotropic increase, normal pressure increase and dyspnoea at 100 W workload. 24-hour Holter-ECG was performed showing 630 isolated premature ventricular complexes (PVC) with two prevalent morphologies and often early, not described in previous Holter-ECGs (figure 2).

**Figure 3:**
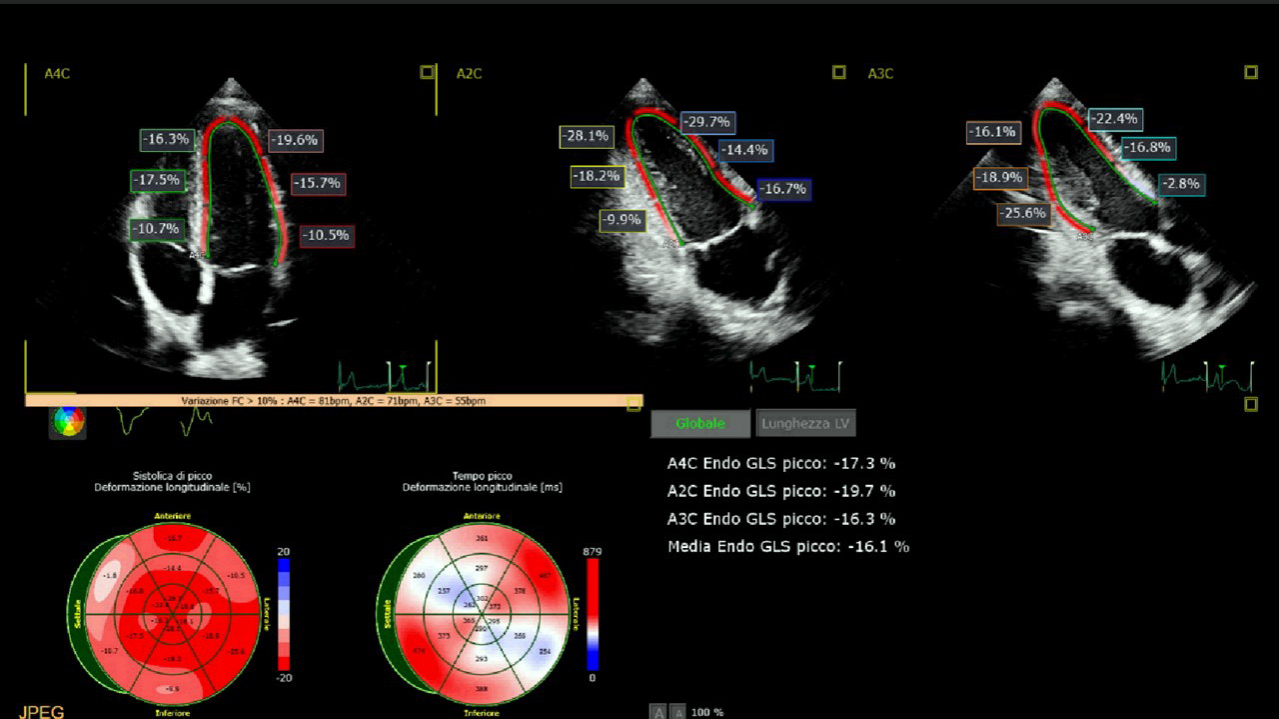
Echocardiographic analysis by speckle tracking: a global longitudinal strain (GLS) of −16.1% is seen.

Echocardiographic examination showed preserved left ventricular systolic function (LVEF) in the absence of segmental dyssynergies, normal pericardium and right sections’ findings (LVEF Simpson method 62% - E' 12 - E/E' 10 - TAPSE 23) and a reduced global longitudinal strain (GLS: −16.1%; figure 3).

Considering the referred symptoms and easy straining, although common symptoms in the post-COVID period, we performed a cardiopulmonary exercise test (CPET) that showed an early anaerobic threshold and a normal peak PVO_2_ (21 mL/kg/min) not in line with the presumed range for a top-level athlete.

**Figure 4:**
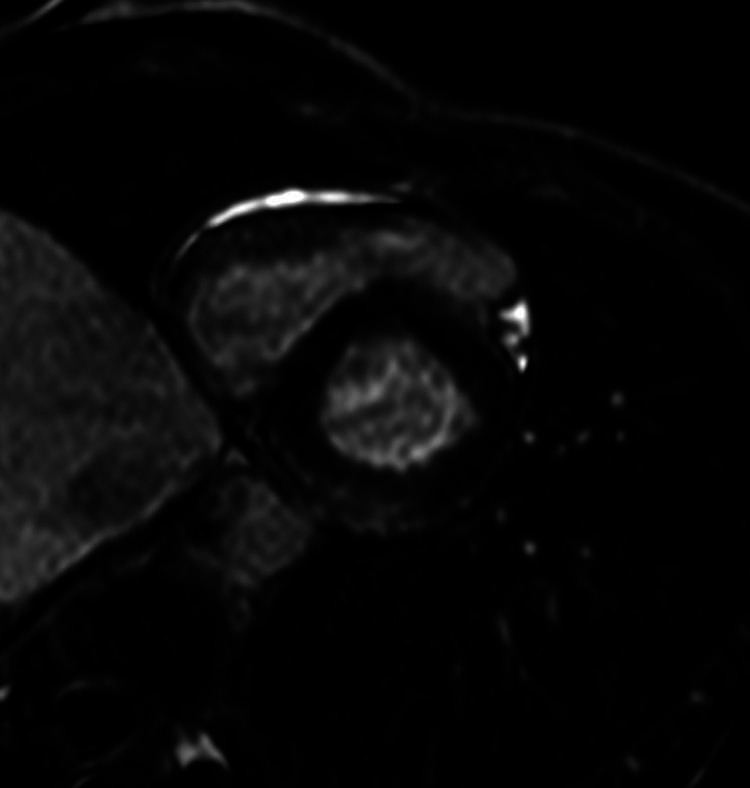
MRI image analysis. Precontrast images: on STIR T2w image areas of signal hyperintensity indicative of myocardial oedema in mid-basal region of inferior and posterior walls are observed.

**Figure 5:**
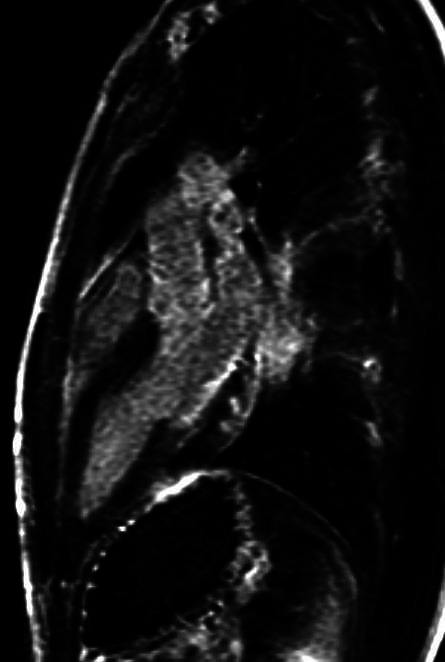
MRI image analysis. Postcontrast images: late images, evidence of enhancement areas with subepicardial/intramyocardial distribution in the basal segments of the inferior wall.

Considering the symptoms described, the CPET data, the evidence of early PVC and GLS reduction, despite the negative first-level investigations, the patient underwent MRI, which documented a myocardial oedema in the mid-basal region of inferior and posterior walls of the left ventricle (figures 4 and 5).

## Treatment

Suitability for competitive practice was suspended, intense isometric efforts were not recommended, a gradual physical readaptation path was started, and therapy with low-dose bisoprolol was set. These precautionary measures were adopted despite it being known that the absence of inflammatory septal involvement generally implies a more benign prognosis.^7^

## Outcome and Follow-up

**Figure 6:**
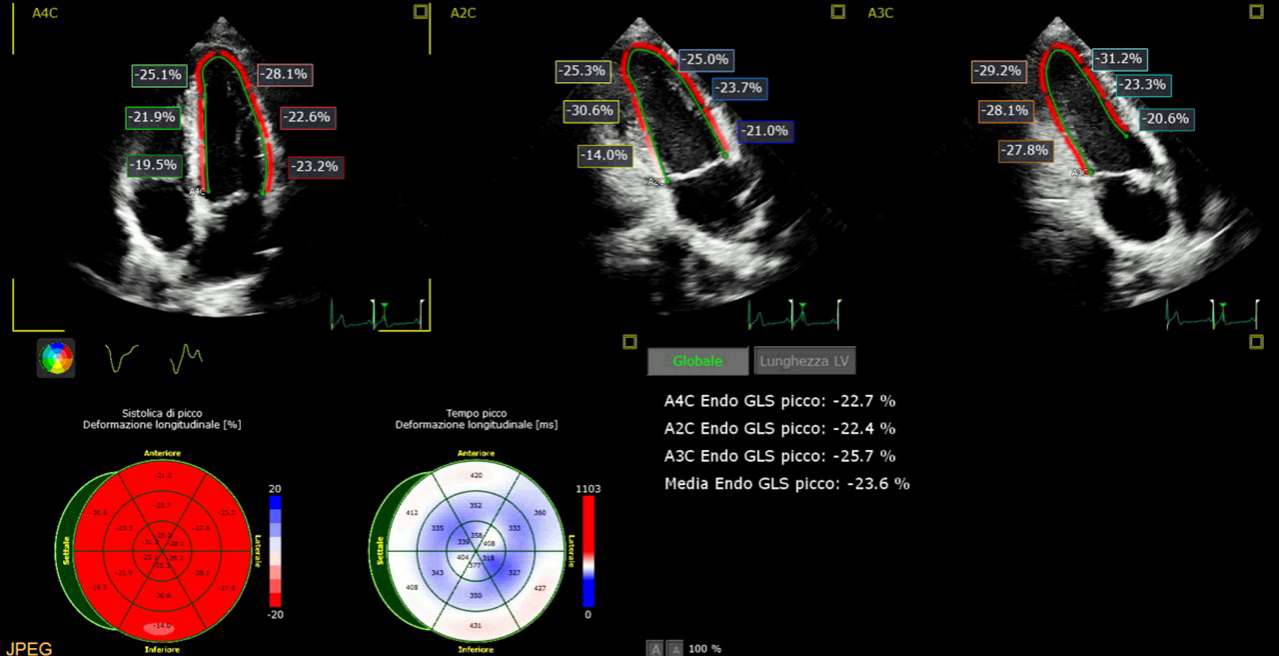
Echocardiographic analysis by speckle tracking: global longitudinal strain (GLS) control 6 months after diagnosis (−23%).

The patient returned to our attention 6 months later. Gradual clinical improvement was reported during follow-up. The echocardiographic examination by speckle tracking analysis showed a GLS normalisation (GLS −23% - LVEF 64% - E' 15 - EE/E' 8; (figure 6).

The ergometric test with cardiopulmonary analysis showed a realignment of the HR/VCO_2_ curve with a late threshold and increment of peak PVO_2_ (39 mL/kg/min).

**Figure 7:**
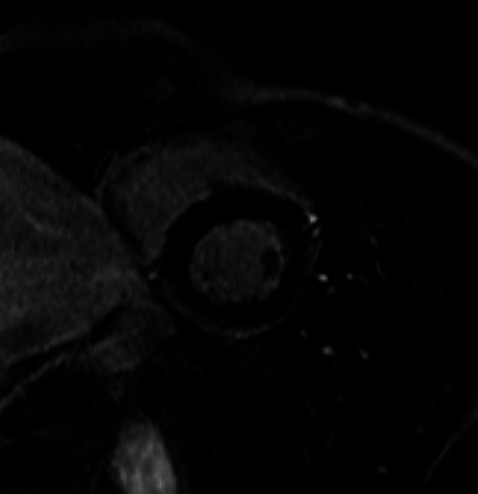
MRI analysis. Precontrast images: on stir T2w images, absence of myocardial areas with signal hyperintensity compatible with oedema.

**Figure 8:**
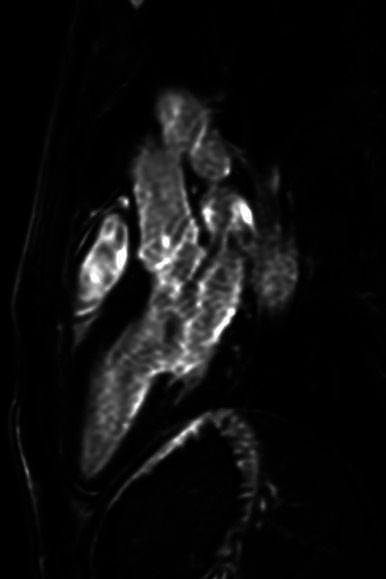
MRI image analysis. Postcontrast images: on late images, the presence of enhancement areas with lower basal distribution compatible with myocardial fibrosis.

The 6-month control MRI examination showed almost no signs of myocardial inflammation and, for this reason, the patient was declared suitable for returning to competitive sport (figures 7 and 8).

## Discussion

It is now known that COVID-19 infection can cause inflammatory damage to the heart muscle.^1 2^

This damage can be explained both as a widespread inflammatory damage typical of inflammatory states in response to viral or bacterial infections, and as an expression of the direct damage of the virus on cardiomyocytes.

To support the first hypothesis, several studies such as Basso *et al*^8^ have shown a lymphocyte infiltration typical of the diffuse inflammatory state in deceased patients with COVID-19 infection. Alongside these studies, several others such as Amendola *et al*^9^ have shown virus direct damage on cardiomyocytes and that this damage correlates with ACE receptor cellular expression.

In a large registry conducted in the USA on collegiate youth with COVID-19 infection, who underwent MRI and other more or less consecutive approaches, a much greater accuracy was shown in defining myocardial events when MRI was used compared with standard approaches.^10^

The MRI examination has a considerable cost, and it is not easily or at least readily usable, especially within a process of investigations such as that established for the release of suitability for competitive sports practice.

Several studies have shown that even in paucisymptomatic patients, the echocardiographic parameter obtained by speckle tracking analysis and especially with the GLS was sensibly reduced compared with the general population.^6^

The reduction of the GLS has been included in several predictive scores useful for guiding the use of MRI investigation in the suspicion of myocarditis.^11 12^

In our case first-level investigations did not support any hypothesis of myocardial inflammation. The basal ECG did not show alterations typical of myocarditic states.^13 14^

The presence of stress symptoms confirmed by CPET data, even if it is a common condition in the post-COVID period, the evidence of often early PVC, not frequent but not mentioned in previous Holter-ECGs, and the very severe GLS reduction led to conduct a deepening investigation with MRI that confirmed the presence of myocardial inflammatory lesion. The 6-month follow-up GLS analysis showed complete normalisation, and this was paralleled by the normalisation of MRI data, thus possibly confirming the appropriateness of the diagnostic approach used.

Our case suggests that within a diagnostic procedure preparatory to the release of the suitability for competitive practice of athletes with recent COVID-19 infection (return to play protocol), the analysis of LVEF by GLS may represent an additional useful element to be considered, in support of standard investigations. Appropriate studies are needed to identify possible benefits of this approach.

**Learning points**

- COVID-19 infection produces myocarditis even if the clinical manifestations are modest, and often the first-level instrumental investigations fail to identify them.
- The myocarditis event in the population of competitive athletes can represent a clinical entity that can sometimes lead to relevant consequences such as unexpected arrhythmic events.
- Echocardiographic analysis with the global longitudinal strain could play an important role in a multiparametric diagnostic evaluation to be reserved for athletes after COVID-19 infection.
